# End-of-life care needs in cancer patients: a qualitative study of patient and family experiences

**DOI:** 10.1186/s12904-024-01489-1

**Published:** 2024-06-21

**Authors:** Mario López-Salas, Antonio Yanes-Roldán, Ana Fernández, Ainhoa Marín, Ana I. Martínez, Ana Monroy, José M. Navarro, Marta Pino, Raquel Gómez, Saray Rodríguez, Sergio Garrido, Sonia Cousillas, Tatiana Navas, Víctor Lapeña, Belén Fernández

**Affiliations:** Asociación Española Contra el Cáncer, Madrid, Spain

**Keywords:** Palliative care, Barriers, Facilitators, Unmet needs, Cancer patients, End-of-life

## Abstract

**Background:**

Cancer is a disease that transcends what is purely medical, profoundly affecting the day-to-day life of both patients and family members. Previous research has shown that the consequences of cancer are greatly aggravated in patients at the end of life, at a time when they must also grapple with numerous unmet needs. The main objective of this study was to obtain more in-depth insight into these needs, primarily in patients with end-stage cancer nearing death.

**Methods:**

Semi-structured interviews were conducted in Spain with cancer patients at the end of life (*n* = 3) and their family members (*n* = 12). The findings from the interviews were analyzed using qualitative thematic analysis and a grounded theory approach.

**Results:**

Four major themes emerged from the interviews that explored the needs and concerns of patients with cancer at the end of life: (1) physical well-being (2) emotional well-being (3) social well-being and (4), needs relating to information and autonomous decision-making. The interviews also shed light on the specific needs of family members during this period, namely the difficulties of managing increased caregiver burden and maintaining a healthy work-life balance.

**Conclusions:**

A lack of support, information and transparency during a period of immense vulnerability makes the end-of-life experience even more difficult for patients with cancer. Our findings highlight the importance of developing a more in-depth understanding of the needs of this population, so that informed efforts can be made to improve palliative healthcare and implement more comprehensive care and support at the end of life.

**Supplementary Information:**

The online version contains supplementary material available at 10.1186/s12904-024-01489-1.

## Background

The concept of end of life reflects both the irreversible progression of a life-limiting disease, and a life expectancy of six months or less [1]. Cancer patients in the end-stage of the disease, facing end-of-life issues, undergo significant physical, psychological and social alterations, as do family members, whose lives and environment are drastically changed during this period. It is therefore of vital importance to improve the quality of life of all of those affected by this diagnosis. During this stage, numerous needs emerge regarding the care and well-being of the cancer patient and their caregivers which are not always acknowledged or identified. Recognition and awareness of these needs can ensure early access and comprehensive treatment for cancer patients with palliative care needs, while also meeting the needs of their caregivers [2, 3].

In regard to patients, the literature provides some insight into the needs that emerge at this stage of the disease, yet there are still many areas that require further investigation. In a review by Wang et al. [4], which analyzed patients’ unmet needs in 38 studies, the authors identified seven major categories of needs. These included physical needs and symptom control, the need to maintain functionality and day-to-day activities, the need for information, psychological needs, social and financial needs, and spiritual needs. The relationship between various sociodemographic variables and patients’ unmet needs has also been studied. Several studies found that female patients had a higher frequency of unmet physical and psychological needs than male patients [5, 6]. Similarly, patients with a high education level reported more unmet needs in physical domains, functionality, day-to-day activities [7] and information [8]. In addition, social needs appeared to a lesser extent in high-income patients [9]. Severe symptoms of emotional stress, the presence of anxiety and/or depression, a greater lack of problem-focused coping, and poorer quality of life of caregivers were identified as negative predictors of patient satisfaction [10]. Finally, when regarding primary caregivers, patients reported more unmet needs when their caregivers were male, young, or showed high levels of emotional distress [6].

For informal (or family) caregivers, the supportive care process is physically and psychologically challenging, especially when caring for patients with advanced cancer at the end of life [11]. However, assessing the unmet needs of caregivers remains an uncommon practice. Many family members, including those who do not view care giving as a burden, suffer from a wide range of problems, including sleep alterations, anxiety, depression, difficulty balancing caregiving and daily tasks, and financial burdens [12]. Wang and colleagues [4] found that caregivers had unmet needs regarding information about the disease and its treatment [11, 13–16], as well as psychological [17, 18], economic [19] and spiritual needs [20]. Researchers also reported that younger caregivers had a higher number of unmet needs than older caregivers regarding care, information and economic needs [13, 14]. Caregivers with physical problems experienced a greater number of unmet needs. Similarly, family caregivers reported more needs when patients suffered from anxiety, depression, or poor physical performance [14]. Family members also experienced a need for clear and reliable information that would help them prepare for their loved one’s death and the grieving process [21, 22].

There is a genuine necessity for studies that integrate patient and caregiver needs, in part to illustrate and conceptualize a unique family unit, given that the different elements that comprise this unit interact and impact on one another. The literature clearly shows that the unmet needs of patients can increase the physical and emotional burden of the caregiver [23]. In turn, caregiver issues are closely related to patient well-being [24]. The unresolved issues or unmet needs of caregivers will not only diminish their own quality of life, but also negatively impact on patients’ health outcomes [25].

Finally, it should be noted that few studies focus on the changing needs of patients and family members at the end of life, or of bereaved caregivers coping with grief. Our research aims to help fill this gap in the literature, using qualitative methodology to identify the unmet needs and potential barriers [3, 26–28] encountered by these patients and their families, and thus provide greater insight into what the end of life is, as opposed to what it could and should be, for all those involved.

## Methods

### Design

An exploratory descriptive qualitative study was carried out using semi-structured interviews with patients with end-stage cancer at the end of life and their family members. Fieldwork was conducted in May and June 2023. The Charmaz grounded theory approach to thematic analysis was implemented [29, 30]. Categories of analysis have been generated inductively and deductively, following a grounded theory approach, and then grouped into themes. This methodology allows researchers to delve into areas of knowledge and reality from a novel perspective, making it possible to explore common perceptions and experiences as part of the subjective social structure of the participants. The study was designed and developed following the consolidated criteria for reporting qualitative research (COREQ) [31].

### Participants

In this study, purposive sampling was used to identify participants, and obtain the maximum variation of the designed sample. This type of sampling is a strategy used to gather participants in a given context with expertise in the study phenomenon. Participants were recruited by health and social professionals through an association of cancer patients. The health professionals in direct contact with the participants informed them about the study and its objectives. Subsequently, and after signing the written informed consent sheet, the research team contacted the participants directly via e-mail or telephone call to confirm the interview. From an initial contact with 35 participants, the research team finally conducted 11 interviews with 15 participants. The variables chosen for the participants were: phase of the disease/after death (advanced, end of life, recent bereavement, bereavement after 6 + months), place of residence (rural, urban), age (< 65 or > 65) and gender. The sample also had ample experience with palliative care services, both at home and in hospital outpatient visits.

The final sample included 15 participants: 3 patients with end-stage cancer diagnosis and 12 caregivers who cared for terminally ill family members and experienced bereavement. A total of 10 semi-structured in-depth interviews were conducted, two of them in pairs (patient and relative), and another in a group composed of three caregivers. By including relatives of different ages, marital statuses and roles within the family, different narratives can be explored through the diverse perspectives, conflicts and connections collected.

Initially, 16 participants were proposed for the sample. However, theoretical saturation was reached at 15 participants, referring to the point in the research where new interviews added nothing novel or relevant to the study objectives [32], resulting in the final inclusion of 15 subjects. Table 1 shows the sociodemographic characteristics of these participants.

**Table 1:** Sociodemographic characteristics of research participants

Participant Code	Participant Profile	Participant gender	Patient gender	Patient age	Patient Status	Phase of disease	Place of residence
P1	Patient	Man	Man	< 65	Independent	End-of-life	Urban
P2	Patient	Man	Man	> 65	Independent	End-of-life	Urban
P3	Relative (spouse)	Woman					
P4	Patient	Man	Man	< 65	Independent	End-of-life	Rural
P5	Relative (spouse)	Woman					
P6	Relative (daughter)	Woman	Man	< 65	Independent	Bereavement after 6 + months	Urban
P7	Relative (son)	Man	Man	> 65	Dependent	Recently bereaved	Urban
P8	Relative (spouse)	Woman	Man	< 65	Independent	End-of-life	Urban
P9	Relative (daughter)	Woman	Man	> 65	Independent	End-of-life	Rural
P10	Relative (son)	Man	Woman	< 65	Dependent	Bereavement after 6 + months	Urban
P11	Relative (spouse)	Man	Woman	> 65	Dependent	End-of-life	Rural
P12	Relative (daughter)	Woman	Woman	< 65	Dependent	Recently bereaved	Urban
P13	Relative (spouse)	Woman	Woman	< 65	Dependent	Bereavement after 6 + months	Rural
P14	Relative (spouse)	Woman	Man	> 65	Dependent		Urban
P15	Relative (spouse)	Man	Woman	< 65	Dependent		Urban

### Data collection

All interviews were conducted online through a videoconferencing platform, except for two that were conducted by telephone at the request of the interviewees. They were conducted by two professionals with experience in qualitative research on healthcare and palliative care who had no previous contact with the participants in order to avoid bias in data collection. The interviews were based on a broad directive script, designed by a team of oncology psychologists and social workers with extensive experience in palliative care. This script was adapted to the context and profile of each person that was interviewed. Family caregivers were asked about the needs of the patients and as well as their own needs. The main topics discussed were: (1) Perceptions about end-of-life and palliative care; (2) Difficulties in the end-of-life process and the needs detected, both met and unmet; (3) The demands and proposals at professional and institutional level for the resolution of these needs (see Supplementary Materials). Interviews lasted an average of 45 min.

The interviews were audio recorded with the consent of the participants, and later verbatim transcribed and anonymized in their entirety. Written informed consent sheet was provided to participants by the professionals who made the recruitment. In addition, the researcher who conducted the interviews asked for oral consent to the participants right before the interviews. They were also provided the opportunity to ask questions and dispel doubts before starting the interview process. No payment was offered in exchange for participation in the study. Personal data and digital rights of participants were protected in accordance with the LOPDGDD 03/2018 [33].

### Data analysis

To get an overview of the information before starting with the initial coding, 5 researchers of the team read the entire transcriptions repeatedly. The transcripts were then segmented into text units relevant to the analysis, which were subsequently read by all researchers. Finally, segments from the interviews were coded based on degree of relevance. A code tree was produced, combining closed categories previously discussed by the research group with categories that emerged inductively from the collected data. This tree was tested in the coding of certain interviews and adapted throughout the field research process to improve its scope and effectiveness. Data collection and analysis occurred concurrently. The information from the codes generated was compared with each other in order to create new categories. The codes were analyzed using both an inductive and a deductive approach that identifies similar, interrelated patterns and allows the theoretic categories described in the study to emerge from the raw data itself. Analytical rigor was assured through a rigorous process of peer review, since other researchers from the team were invited to review the initial analysis, in order to confirm the consistency of the findings and to identify and diminish potential biases. All the researchers agreed with the results of the coding process and the generation of the main categories. Data analysis was performed using ATLAS.ti version 9.

## Results

The results were divided into two main sections: the needs of people diagnosed with cancer at the end of life and the needs of family caregivers during this final phase of care.

### End-of-life needs for cancer patients

The main results are shown in Table 2. The needs of these patients are modified in accordance with disease progression and proximity to death.

**Table 2:** Analysis of Patient Needs Outcomes

**Patients**	**Topics**	**Needs**
	Physical well-being	Symptom monitoring and control
		Sleep & Rest
		Cleanliness and personal hygiene
		Nutrition
		Physical activity
	Emotional well-being	Tracking emotional well-being
		Addressing Emotional Distress
		Seeking out distractions
		Self-identity and self-esteem
		Affective relationships
		Social Relationships
	Social well-being	Cover costs of technical and/or orthotic and prosthetic devices
		Cover costs of professional care
		Personal space
		Domestic mobility
		Transfers by vehicle
	Information and autonomy in decision-making	Reliable and transparent information
		Patient’s wills and preferences

#### Physical well-being

One of the main themes that emerged from participant responses was the need to maintain a sense of physical well-being. This set of needs is aimed at improving their quality of life by monitoring the symptoms of both the disease and its treatment. The main codes indicated by participants were the need to control pain and the adverse effects of medication, to find cures and clinical procedures to ensure quality of life, and to organize the administration of medication.

*“That I, her daughter, have to act as a nurse, as I watch my mother dying and being told on the phone: ‘inject this, inject that’.” P12*.

The other codes regarding physical well-being were functional problems related to care. These needs include optimal rest and sleep, adapting diet and eating habits to allay weight gain or lack of appetite, and maintaining personal hygiene and physical activity.

*“Well, you have to be aware of the circumstances. In terms of strength, it depends on the day…I’ve lost a lot of weight and I don’t sleep well, but I used to sleep very well. And this, of course, limits you.” P1*.

*“I have my doubts about what the best diet is. The oncologist doesn’t know. I found a nutritionist, and it’s a little clearer now, but I think I lack information. Nothing is said about the diet…” P1*.

#### Emotional well-being

Another theme that concerned participants was their emotional well-being. Participants put a high priority on addressing emotional needs related to illness, dependency and death, as well as having access to assistance in effectively expressing and communicating these needs.

*“Nobody talks about the psychological burden that this disease can place on the patient. How your life changes, all that it entails. The pain, going to the hospital continuously, and not knowing what will happen in the near future. More help is needed so we can understand how to relate to the disease rather than confront it.” P1*.

Participants also reported the need for the care and protection of their of identity, of their roles and purpose in life, as well as their self-image and self-esteem, all of which are frequent concerns of people facing the end of life.

*“So it was very difficult for him … to be aware, and lose those roles and lose the role of doctor, lose the role of father, lose the role of person… in other words, to be sick person.” P6*.

Other relevant codes included the need to maintain affective relationships by spending quality time with your loved ones, and getting support to carry out fulfilling activities, hobbies and everyday pleasures.

*“I brought her home, with her granddaughter, who she was mad about, and my kitten. My mother has always loved my cat. When I brought her home on the first day in the wheelchair, the cat climbed onto her lap and gave her a kiss. And now, on her deathbed—the last days of her life—my cat will not leave her side.” P12*.

#### Social well-being: adapted living space and mobility and economic resources

Most of the participants reported that a well-adapted living space was essential for providing comfort and quality of life at the end of life. This corresponds to the need to adjust the living environment to the patient’s physical circumstances during the end-stage of the disease.

Participants prioritized the following codes: the need for material resources that could improve the patient’s quality of life (medical equipment and supplies, hygiene products, etc.), domestic mobility (wheelchairs, stair lifts, etc.), and comfortable living spaces adapted to the patient’s physical needs.

*“Now we must hustle and bustle with a wheelchair, because I cannot walk. We requested a wheelchair and the doctor prescribed it for me, an electric wheelchair. They prescribed it for me, they accepted it and today a letter has come, saying it has been denied.” P2*.

*“I have a bathtub and I couldn’t afford it… Also, I pay rent, so I couldn’t replace the bathtub with a shower. I had no time, no disposition, no money. I ultimately had to buy an orthopedic chair so I could shower her in the bathtub” P12*.

Another important aspect of social well-being highlighted by the participants was the need to have access to professional resources that cover daily tasks, mainly through the hiring of professional caregivers.

*“And the aid they gave was quite small, that’s also something that could, that should be considered, right? Above all, people who need care, a lot of care. […] In the end, it’s true that you need 24 hours, 7 days a week.” Family Group, P14*.

#### Information and autonomy in decision-making

Needs related to informed decision-making are associated with a willingness to ensure patient inclusion and autonomy in decisions that are made throughout the end-of-life period. The interviewees emphasized that they often felt as if healthcare professionals were not honest and forthright when sharing information with them. Yet they need this information to cope with doubts and make informed decisions, information on the prognosis of their disease and therapeutic options, legal and bureaucratic procedures, psychological and social resources, and shared experiences of other patients. In addition, they expressed the need for the patient’s will and preferences to prevail.

*“Learn more about the process. Like: “The degeneration will progress more and more.” Yes, but how will this degeneration take place? What exactly should I expect? I mean, I know they can’t give me a timeline, I understand that. Learn how the disease really works. You search the Internet, and then have to hope that your search is right.” P9*.

### End-of-life needs for family caregivers

The daily tasks of a family caregiver together with the challenges that arise during end-of-life care for a cancer patient can overwhelm the caregiver. In most cases, caregiver overload causes severe stress and physical, emotional and mental exhaustion. Family caregivers must learn to balance caring for their loved ones with caring for themselves. This includes organizing work, home, and leisure activities, improving time management, and building a strong internal support system. Family member interviews also revealed a need for greater awareness of their own specific needs, which are often overlooked. These main results are shown in Table 3.

**Table 3:** Results of the analysis of family caregiver needs

**Family Caregivers**	**Topics**	**Needs**
	*Personal health and well-being*	Personal health and physical well-being
	*Emotional Well-Being*	Emotional well-being
		Managing responsibility of caregiving
		Managing fear of losing a loved one and coping with grief
	*Caregiver overload and work-life balance*	Disconnect and rest
		Work-life balance
		Support and assistance in caregiving

#### Personal health and well-being

Family caregivers reported that they often overlooked their health, neglecting their nutrition and adopting inadequate sleep patterns. Those suffering from an illness also noted greater neglect of their own medical needs and treatment.

*“From the time I woke up at 7:00 or 8:00, I didn’t stop, until I went to bed at 12:00. I’ve already lost a lot of weight from the stress. I’ve always said that I have to take care of myself, that I can’t be foolish and neglect myself, because if I fall, we all fall and I have 3 people under my care.” P12*.

#### Emotional well-being

Caregivers in the study also highlighted the need for assistance in minimizing uncertainty and emotional distress. They reported feeling overwhelmed with their caregiving responsibilities, which brought on more pressure and feelings of guilt.

Other emotional needs included managing their fear of losing a loved one and coping with grief and life changes after the death of the patient.

*“I had prepared a letter for my father, because I hadn’t been capable of saying goodbye yet.” P6*.

#### Caregiver overload and work-life balance

The main difficulty that family caregivers faced was achieving a healthy balance between work and personal life. The combination of working and family responsibilities (of both the patient and other family members) can virtually do away with a caregiver’s rest and free time.

*“My father was on medical leave because of anxiety, until my mother died and then he went back to work. Until it happens to you, you don’t realize how much a person needs to stop working, and that it’s not a whim. In other words, you either stop working or caregiving.” P10*.

The participants emphasized the need for caregivers, in this case, mainly family members. Great importance was given to the possibility of having a formal and informal support network, especially at an emotional level and also to collaborate with daily caregiving tasks.

“*The end was appalling, especially because of the work-life balance. It was very burdensome to have the illness at home. I managed to do it because my father was there, otherwise I wouldn’t have been able to go out […] because I couldn’t disconnect at home*.” *P10*.

## Discussion

The objective of this study was to explore the needs of people with cancer at the end of life and the needs of their family caregivers. Its novelty lies in its ability to provide relevant information on the unmet needs of these individuals in a qualitative manner during the end-stage of the illness.

There is limited research on this period given the complexities of reaching out to the cancer population and their loved ones at such a difficult time in their lives. Furthermore, there is the complex task of incorporating the experiences of both patients and family members into a single study and viewing them as a unit that interconnects with the social, economic and institutional reality in which they live.

Based on the needs expressed by the participants, our findings suggest that numerous unmet needs may exist among cancer patients and informal caregivers during the end-of-life period. The complexity and magnitude of the challenges they face during this phase of cancer contribute to a significant array of needs. Although there is diversity in the identified needs among patients and family caregivers, there are also a remarkable number of these needs overall. These findings are consistent with results from similar studies conducted on cancer patients in other countries during earlier phases of the end of life.

In terms of the physical well-being of the cancer patient, our findings support other studies in which the need for symptom control, mainly pain and fatigue [5, 13, 34–37], is considered a priority. Another primary concern is the need for rest and adequate sleep. It should be noted that while caregivers usually attend to the needs of the patient regarding nutrition and hygiene, most of them express a sense of over-delegation when it came to these tasks, as well as a lack of preparation for carrying them out.

The need for emotional well-being is of particular concern to the interviewees, constituting one of the primary sources of vulnerability at the end of life. This phase of the disease is characterized by fear and uncertainty and the need to come to terms with impending death. Priority is given to managing emotions related to illness, dependency, dying and death. Patients also stress the importance of spiritual needs during this period, as they face end-of-life issues and reflect on the purpose and meaning in their lives. Preserving one’s identity, as well as one’s self-image and self-esteem also contributes to emotional well-being for people nearing the end of life. These findings support previous research where patients expressed a fundamental need for managing psychological distress [6, 7]. A systematic assessment of the emotional needs of these patients would help identify appropriate coping strategies to reduce emotional distress and lessen its burden on caregivers.

Participants in our study, as in other research, emphasize the unmet need for information that would help them make decisions and express their preferences for end-of-life care [7, 8, 13], thereby ensuring control over their care while easing the burden of decision-making for their caregivers. These findings highlight the need to improve communication between patients and their healthcare providers. By learning and responding to what their patients want to know, these professionals can improve the patient’s understanding of their circumstances and the options available to them at this critical period in their lives.

Regarding social needs, our data show that a well-adapted living space that provides comfort and mobility is vital for dignity at the end of life, as is being able to choose to stay at home rather than move to a hospital. Participants indicated that the main barriers to satisfying these needs were a lack of economic resources to refurbish their home or obtain technical and/or orthotic and prosthetic devices (wheelchair, adjustable beds, etc.) to improve their quality of life. It is necessary to provide assistance and information on adapting the home and devices that facilitate the patient’s mobility. Similarly, more information should be made available regarding economic aid or funds that may be requested to offset these costs and the loss of income resulting from the illness.

Finally, findings on the needs of family caregivers show the challenges that arise during end-of-life care for a patient with cancer cause an overload, which in most cases leads to severe stress and physical and psychological exhaustion. Family caregivers need to learn how to improve their work-life balance. This includes organizing work, home, and leisure activities, improving time management, and building a strong internal support network.

## Conclusion

This study contributes to the relatively small number of publications on patients with cancer and their caregivers at the end of life. In addition, this is the first study carried out in Spain that explores the needs of cancer patients in palliative care and their families based on their own personal experiences. The primary strength of our research lies in the inclusion of end-of-life patients nearing death who had diverse experience with the services provided during end-of-life care. Our main findings also corroborate previous research, while providing a more in-depth understanding of these patients’ needs and their connection to the needs of family caregivers. However, while it was possible to saturate the resulting codes theoretically and empirically, our limited sample size prevented the findings from being generalizing. Nevertheless, these results support the growing call for a more in-depth understanding of the needs of this vulnerable population. Only with this first-hand knowledge any real and effective change can be made to improve palliative healthcare and implement more comprehensive care and support at the end of life.

### Electronic supplementary material

Below is the link to the electronic supplementary material.

Supplementary Material 1

## Data Availability

The dataset generated and analyzed during the current study is not publicly available because individual privacy could be compromised but is available from the corresponding author on reasonable request.
