# Supplementary material for: End-of-life care needs in cancer patients: a qualitative study of patient and family experiences
Source: BMC Palliat Care. 2024 Jun 21;23:157. doi: 10.1186/s12904-024-01489-1 (PMC11191331; doi:10.1186/s12904-024-01489-1)
Supplement: Supplementary file 1 — Supplementary Material 1 [file 12904_2024_1489_MOESM1_ESM.docx]

**Supplementary Material**

**Main Interview Topics**

1. **Interview Framework**

- Presentation of the study and its objectives.
- Preparation of the topic to be discussed.
- Explanation of the dynamics of the interview process.
- Ethical aspects.

1. **Perceptions of the end of life and palliative care**

- Exploration of previous concepts regarding death and the end of life and their connection to current perceptions and attitudes.
- Perception and assessment of palliative care. Knowledge and experience.
- **Difficulties during the end-of-life period and identified needs**
- General exploration of perceived needs.
- Specific exploration of needs. *If they do not arise spontaneously, the following needs will be explored:*
  - Physical, pharmacological, and medical needs.
  - Needs related to mental and emotional health.
  - Affective needs.
  - Social needs.
  - Spiritual needs.
  - Material or resource needs.
  - Information and decision-making needs.
- Once needs and difficulties are identified, the following aspects will be explored:
  - Whether or not they are resolved, who resolves them, and how.
  - Who do you think should resolve them and how?
- Explore examples of met and unmet needs/difficulties.

1. **Demands and suggestions**

- Identification of demands for palliative care at the professional and institutional level.

1. **Closure**

- Farewell and gratitude to the participants.
